# Contribution of basal ganglia activity to REM sleep disorder in Parkinson’s disease

**DOI:** 10.1136/jnnp-2023-332014

**Published:** 2024-04-19

**Authors:** Zixiao Yin, Tianshuo Yuan, Anchao Yang, Yichen Xu, Guanyu Zhu, Qi An, Ruoyu Ma, Yifei Gan, Lin Shi, Yutong Bai, Ning Zhang, Chunxue Wang, Yin Jiang, Fangang Meng, Wolf-Julian Neumann, Huiling Tan, Jian-Guo Zhang

**Affiliations:** 1 Department of Neurosurgery, Beijing Tiantan Hospital, Capital Medical University, Beijing, China; 2 Movement Disorder and Neuromodulation Unit, Department of Neurology, Charité – Campus Mitte, Charite – Universitatsmedizin Berlin, Berlin, Germany; 3 Department of Neuropsychiatry, Behavioral Neurology and Sleep Center, Beijing Tiantan Hospital, Capital Medical University, Beijing, China; 4 Department of Functional Neurosurgery, Beijing Neurosurgical Institute, Capital Medical University, Beijing, China; 5 Medical Research Council Brain Network Dynamics Unit, Nuffield Department of Clinical Neurosciences, John Radcliffe Hospital, University of Oxford, Oxford, UK

## Abstract

**Background:**

Rapid eye movement (REM) sleep behaviour disorder (RBD) is one of the most common sleep problems and represents a key prodromal marker in Parkinson’s disease (PD). It remains unclear whether and how basal ganglia nuclei, structures that are directly involved in the pathology of PD, are implicated in the occurrence of RBD.

**Method:**

Here, in parallel with whole-night video polysomnography, we recorded local field potentials from two major basal ganglia structures, the globus pallidus internus and subthalamic nucleus, in two cohorts of patients with PD who had varied severity of RBD. Basal ganglia oscillatory patterns during RBD and REM sleep without atonia were analysed and compared with another age-matched cohort of patients with dystonia that served as controls.

**Results:**

We found that beta power in both basal ganglia nuclei was specifically elevated during REM sleep without atonia in patients with PD, but not in dystonia. Basal ganglia beta power during REM sleep positively correlated with the extent of atonia loss, with beta elevation preceding the activation of chin electromyogram activities by ~200 ms. The connectivity between basal ganglia beta power and chin muscular activities during REM sleep was significantly correlated with the clinical severity of RBD in PD.

**Conclusions:**

These findings support that basal ganglia activities are associated with if not directly contribute to the occurrence of RBD in PD. Our study expands the understanding of the role basal ganglia played in RBD and may foster improved therapies for RBD by interrupting the basal ganglia-muscular communication during REM sleep in PD.

## Introduction

Rapid eye movement (REM) sleep behaviour disorder (RBD) is characterised by dream enactment behaviour and loss of muscle atonia during REM sleep.^1^ It is a parasomnia strongly linked to the occurrence of Parkinson’s disease (PD) and other neurodegenerative disorders.^2^ The presence of RBD can precede the onset of PD by several years, making it an important feature of prodromal PD.^3^ Currently, the mechanisms underlying the occurrence of RBD in PD are not yet fully understood.

Abnormal neural communication in the basal ganglia circuit is a major pathological manifestation in PD,^4^ but its importance for the occurrence of RBD remains under debate. In the awake state, movements in PD are characterised by bradykinesia and rigidity that have been linked to an imbalance of direct and indirect basal ganglia pathways.^5^ Based on the observation that patients with PD can exhibit a restoration of motor control during RBD,^6 7^ one hypothesis proposed that the basal ganglia loop may be bypassed in the pathophysiology of RBD. However, recent studies provided evidence showing that the basal ganglia beta power and high-frequency activity are significantly modulated by REM sleep without atonia (RSWA) and RBD movements,^8 9^ suggesting that the basal ganglia structures may still be engaged in the development of RBD. Yet currently, a rigorous quantification of both the basal ganglia and muscular activities during REM sleep has not been performed. In addition, a potential relationship between basal ganglia activities and the severity of RBD in PD remains unexplored.

Here, we recorded electrophysiological activity as local field potentials (LFPs)^10 11^ directly from two major basal ganglia structures during REM sleep in two cohorts of patients with PD that underwent deep brain stimulation (DBS) surgery. Given the inherent absence of healthy control groups in invasive neurophysiology studies, we compared basal ganglia oscillatory patterns from PD to an age-matched cohort of patients with another neurological disorder, namely dystonia, which is less frequently associated with RBD.^12^ We identified electrophysiological signatures of basal ganglia activity that are directly correlated with the clinical severity of RBD in PD.

## Materials and Methods

### Patients and surgery

Three cohorts of patients with movement disorders scheduled to receive DBS surgery were included, including patients with PD receiving either globus pallidus internus (GPi)-DBS or subthalamic nucleus (STN)-DBS, and patients with dystonia receiving GPi-DBS. The determination of STN-DBS or GPi-DBS for patients with PD was based on clinical consideration. Patients with levodopa-induced dyskinesia, cognitive decline and psychiatric symptoms were more likely to undergo GPi-DBS. The inclusion and exclusion criteria were detailed in online supplemental materials. We performed DBS surgery as per routine protocol.^13^ Four-contact DBS electrodes were bilaterally implanted under local anaesthesia. Before the implantation of pulse generator, DBS electrodes were externalised for basal ganglia LFP recordings in parallel to the video polysomnography (PSG) during sleep. All patients signed written informed consent.

### Sleep recordings

**Figure 1:**
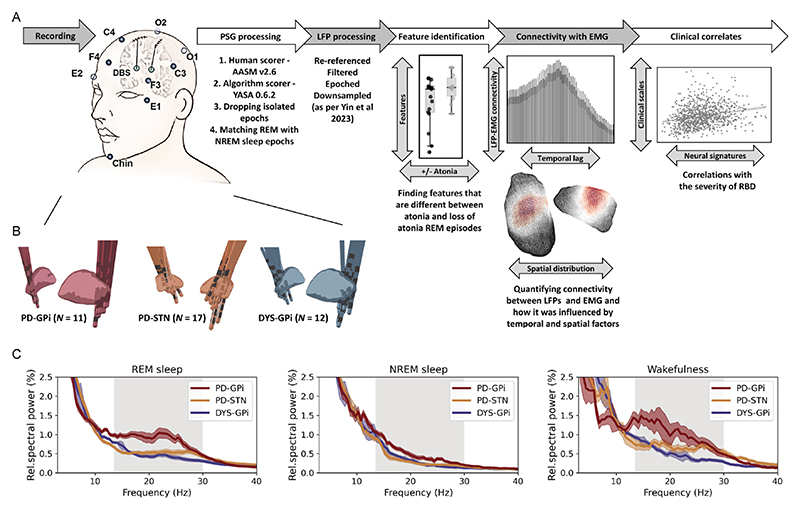
Data recording/processing pipeline and basal ganglia power spectra across groups. (A) Summary of the data recording and processing pipeline. See text for more details. (B) Localisation of deep brain stimulation electrodes in three groups of patients. (C) Average power spectra in different sleep states across three groups of patients. The shaded area indicates SEM. The beta band range is highlighted in grey. EMG, electromyogram; LFP, local field potential; NREM, non-rapid eye movement; PSG, polysomnography; REM, rapid eye movement; RBD, rapid eye movement sleep behaviour disorder.

Sleep recording was performed during lead externalisation 2–5 days after the lead implantation and lasted for 1–2 consecutive nights as previously described.^14^ Medications were stopped during recording (anti-dystonia drugs stopped after midday and anti-parkinsonism drugs stopped after 18:00). See online supplemental table 1 for medication information. Stimulation was kept off throughout the recording period. PSG was implemented including electroencephalogram, electrooculogram, electromyogram (EMG) and video. Basal ganglia signals were simultaneously recorded (figure 1A). All signals were recorded through the JE-212 amplifier (Nihon Kohden, Tokyo, Japan) at a sampling rate of 1000 or 2000 Hz after amplified ×195 and bandpass filtered between 0.08 and 300 Hz.

### PSG processing

The processing of PSG included sleep scoring, epochs dropping and epochs matching. First, for each 30-s PSG epoch, sleep stages including awake, N1, N2, N3 and REM sleep were scored by two experienced sleep specialists based on the American Academy of Sleep Medicine criteria version 2.6. To reduce the staging bias introduced by the subjectivity of human scorers,^15^ PSG data were also staged by an established algorithmic scorer (https://github.com/raphaelvallat/yasa) that is trained and validated on over 30 000 hours of PSG data as shown in the online supplemental file.^16^ Only epochs with consistent scoring results were eligible for further analysis, which account for 87.2% of all epochs. In case of multiple nights were recorded for one subject, only the night with higher counts of REM sleep was further analysed. Second, for a given night, we defined ‘isolated epochs*’* as the epoch that had different stage labels with its neighbouring epochs (eg, a single N2 epoch occurred between two REM epochs). Isolated epochs were dropped from the analysis as they may not represent a stable state of sleep.^16 17^ This led to a further dropping of 5.48% of all epochs. Third, to improve temporal comparability between sleep stages, we matched each REM sleep epoch with its nearest non-REM (NREM) sleep epoch using a without-replacement approach.^18^ Since not all subjects had all three stages of NREM 1/2/3, NREM sleep was treated as one homogenous stage in the main analysis. This procedure resulted in a final amount of 78.0±49.8 REM-NREM epoch pairs for each subject, with an average of 27.8±24.9 min temporal distance between matched REM and NREM epochs. NREM sleep served as the between-stage control of REM sleep. The three substages of NREM were analysed separately in an online supplemental analysis.

### Determination of RSWA

**Figure 2:**
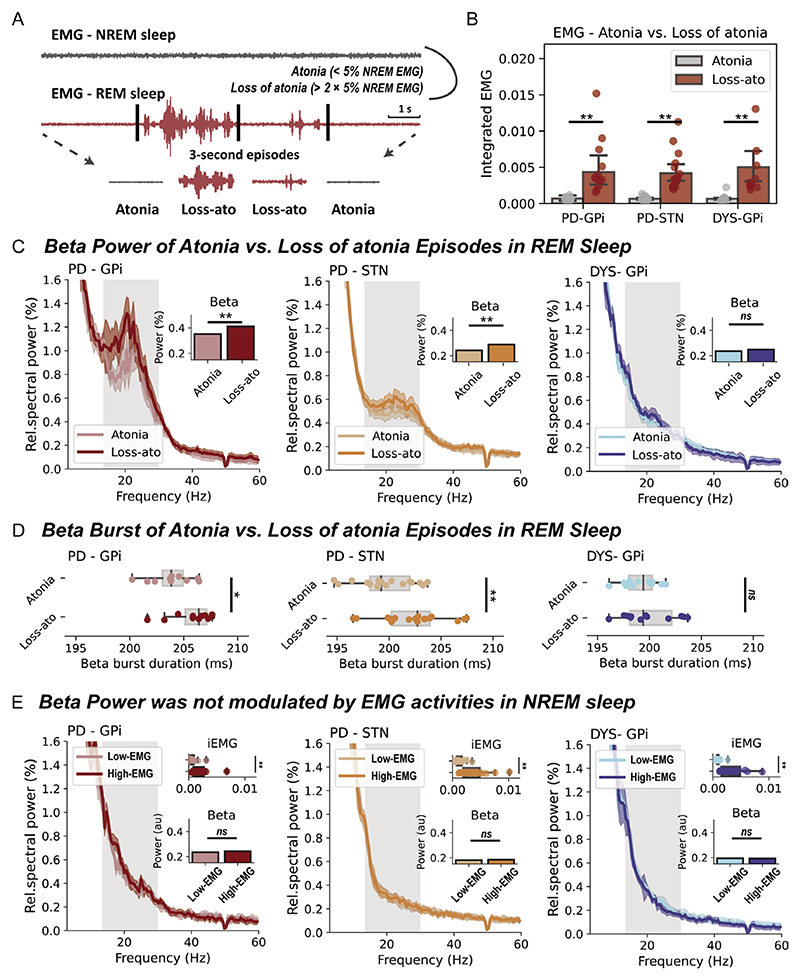
Basal ganglia beta power during rapid eye movement (REM) sleep with and without atonia. (A) A diagram that demonstrates how REM sleep with/without atonia is determined. (B) The integrated electromyogram (EMG) is compared between atonia and loss of atonia REM sleep episodes in three groups of patients. All bar plots indicate mean±SEM. For Parkinson’s disease (PD)-globus pallidus internus (GPi), p=0.002; for PD-subthalamic nucleus (STN), p=7.63×10^−6^; for dystonia (DYS)-GPi, p=4.88×10^−4^, Wilcoxon signed-rank test. (C) The average power spectra and beta power in atonia and loss of atonia REM sleep episodes in three groups of patients. The shaded area indicates SEM. The beta band range is highlighted in grey. **P<0.01. ns, non-significant. Wilcoxon signed-rank test. (D) Beta burst durations in atonia and loss of atonia REM sleep episodes in three groups of patients. For box plots, the lower and upper borders of the box represent the 25th and 75th percentiles, respectively. The centerline represents the median. The whiskers extend to the smallest and largest data points that are not outliers (1.5 times the IQR). For PD-GPi, p=0.042; for PD-STN, p=5.04×10^−4^; for DYS-GPi, p=0.519, Wilcoxon signed-rank test. (E) The integrated EMG, average power spectra and beta power in non-REM (NREM) sleep episodes with below (low-EMG) and above median (high-EMG) EMG activities in three groups of patients. For the comparison of integrated EMG, p=9.76×10^−4^ for PD-GPi; p=1.53×10^−5^ for PD-STN; and p=4.88×10^−4^ for DYS-GPi. For the comparison of beta power, p=0.278 for PD-GPi; p=0.927 for PD-STN; and p=0.791 for DYS-GPi. Wilcoxon signed-rank test. **P<0.01. ns, non-significant.

We determined the presence of RSWA as per previously established approaches.^19^ A REM sleep without muscular atonia threshold was defined as two times of the fifth percentile of individualised NREM chin EMG activity.^19^ For a better temporal resolution, the above judgement was made for each 3-s REM sleep segment (figure 2A).^19^ EMG activities were quantified through the integrated EMG,^20^ which is the area under the curve of the power envelope of filtered EMG signals between 80 and 160 Hz. In addition, a different way of quantifying muscular activity using EMG variance^20^ was also employed when indicated. RSWA episodes have been detected in every recorded patient.

### RBD severity evaluation

The clinical severity of RBD was evaluated subjectively using the RBD-Screening Questionnaire (RBDSQ) before surgery in PD. A further objective evaluation of the RBD severity during the recording night was performed in all subjects based on video-PSG recordings assessing both the movements and vocalisations captured during REM sleep.^21^ Movements were scored between zero to three. Zero, one, two and three points indicated no movement, small movements, proximal movements and axial movements, respectively. Vocalisations were scored between zero to one. Zero and one point indicated no vocalisation and sleep-related sounds other than respiratory noises, respectively. A detailed description of RBD severity for all subjects was provided in table 1.

### Signal processing and spectral analysis

Signals were processed offline using *MNE-Python* and *SciPy* as per previous routines.^14^ Power spectral density was computed between 2 and 60 Hz using the Welch periodogram approach with a Hanning window (1024 samples; 50% overlap) and was normalised to the percentage of sum power. Band power of theta (4–8 Hz), beta (13–30 Hz) and gamma (30–60 Hz) was extracted. From the three bipolar LFP channels from an electrode, the channel demonstrating the highest beta power during wakefulness was selected for all analyses. Basal ganglia-cortex magnitude squared coherence was computed between 2 and 60 Hz using the same Welch’s approach as described in calculating power spectra. For the sake of simplicity, we only computed the coherence between basal ganglia LFPs and ipsilateral central electroencephalogram channels (C3/C4). We extracted the average coherence in theta, beta and gamma bands. The quantification of beta burst dynamics was analysed using previously established approaches and was detailed in online supplemental materials.

### LFP-EMG connectivity

Envelope correlation was employed to investigate the different frequency connectivity between LFP beta power and EMG activities.^22^ LFP and EMG data were first downsampled to 200 Hz and the instantaneous power of LFP beta (13–30 Hz) and EMG (60–90 Hz) activities were extracted through 10-cycle wavelet transforms. The time-resolved power envelope was temporally whitened using a two-order autoregressive model to reduce the impact of autocorrelation on the estimation of envelope correlation.^23^ The Spearman correlation coefficients were averaged first between epochs, then hemispheres and finally subjects. The significance of the empirical coefficient was tested against surrogate coefficients which were obtained by randomly shuffling the time block of the EMG envelope by 200 times. We used the cross-correlation and the Granger causality analysis to investigate the temporal order between LFP beta and EMG activities and used the Lead-DBS toolbox^24^ to conduct spatial localisations of LFP-EMG connectivity. Detailed descriptions of the procedure were shown in the online supplemental materials.

### Statistical analysis

Statistical tests were performed using *Numpy* and *Scipy*. Given the non-parametric nature of most electrophysiological data, we used non-parametric tests throughout this manuscript. These tests included the Kruskal-Wallis test for comparing baseline information among three groups, the Mann-Whitney U test for comparing RSWA and Beta-EMG envelope correlations between patients with PD and dystonia, the Wilcoxon signed-rank test for paired comparisons of integrated EMG, beta power, beta burst duration and G-causality values within groups, and the permutation test for assessing envelope correlation against time-block shuffled surrogates. All results were reported as significant at a two-tailed α level of 0.05.

## Results

This study included 11 patients with PD receiving GPi-DBS, 17 patients with PD receiving STN-DBS and 12 age-matched dystonia patients receiving GPi-DBS. The three groups were comparable in sex (male proportion=45.5%, 58.8% and 50% for the PD-GPi, PD-STN and dystonia cohorts, respectively, p=0.775, Kruskal-Wallis test) and age at surgery (median (IQR)=59.0 (15.0), 64.0 (8.0), and 59.0 (10.5) for the PD-GPi, PD-STN and dystonia cohorts, respectively, p=0.160, Kruskal-Wallis test). Clinical information is shown in table 1. The position of DBS electrodes is shown in figure 1B. Average power spectra across cohorts demonstrated exaggerated basal ganglia beta power (13–30 Hz) in patients with PD across sleep stages, which was more prominent in the GPi than STN, and more prominent during REM and wakefulness compared with NREM sleep (figure 1C). Here, we put our focus on REM sleep.

### Basal ganglia beta power is elevated during RSWA in PD

Patients with PD were associated with significantly higher proportions of RSWA (‘loss of atonia’) episodes than patients with dystonia (PD vs dystonia, 27.0±19.0% vs 7.9±5.8%, p=3.55×10^−4^, Mann-Whitney U test), though in all subjects the integrated EMG was significantly higher in *loss of atonia* than *atonia* episodes (figure 2B). In basal ganglia spectra (figure 2C), beta power was significantly higher in *loss of atonia* episodes in subjects with PD, both for the GPi (p=0.004) and STN (p=3.81×10^−5^, Wilcoxon signed-rank test), but not in subjects with dystonia. Further burst dynamic analysis indicated that the increase of beta power in *loss of atonia* episodes was accompanied by prolonged burst durations (figure 2D).

To investigate whether the observation is specific to beta power, we extended the analyses to other oscillatory features including theta and gamma power, and basal ganglia-cortex coherence. None of these additional features were different between *loss of atonia* and *atonia* episodes during REM sleep in PD (online supplemental table 2).

Furthermore, to investigate whether the observation is specific to REM sleep, we repeated the above analyses in NREM sleep, using median EMG activity to dichotomise NREM sleep into low-EMG and high-EMG episodes. Beta power during NREM sleep was not found to be modulated by muscular activities in any of the studied cohorts (figure 2E). This result remained valid when analysing NREM1/2/3 stages separately (online supplemental figure 1).

### The relationship between basal ganglia beta power and EMG activities during REM sleep

**Figure 3:**
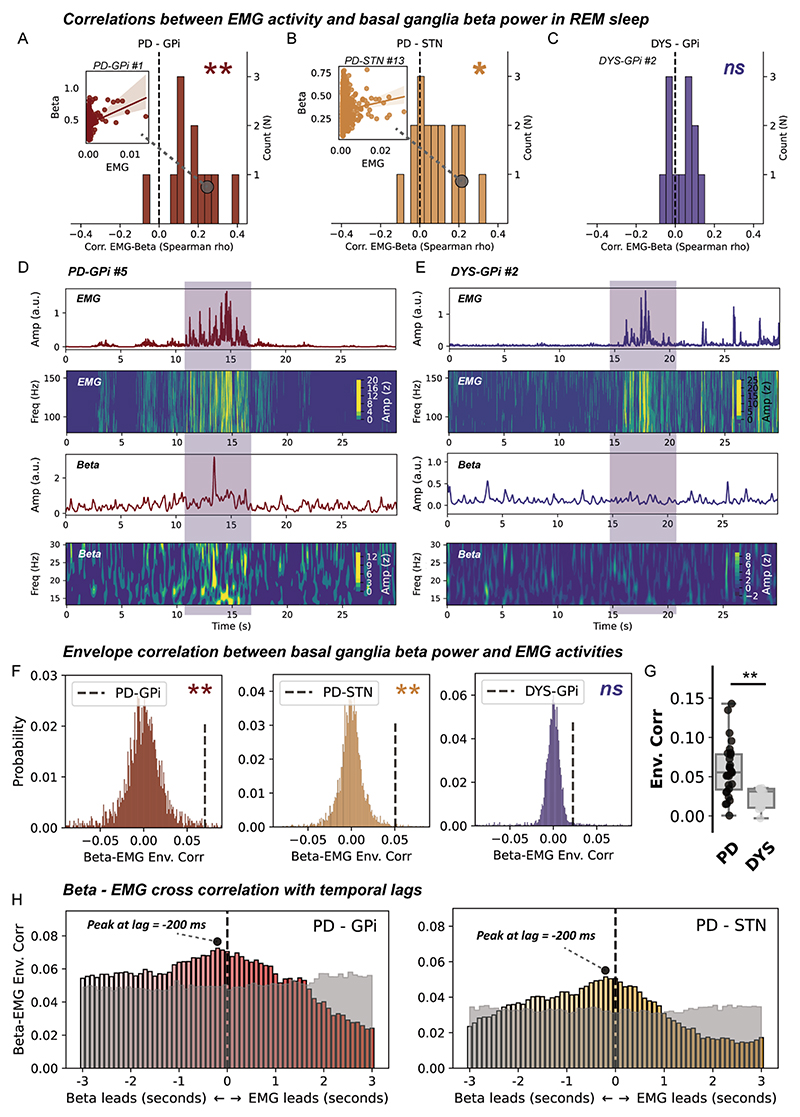
Correlation and connectivity between basal ganglia beta power and electromyogram (EMG) activities during rapid eye movement (REM) sleep. (A–C) The distributions of episode-wise correlation coefficients between basal ganglia beta power and chin EMG activities in three groups of patients. *N* in the y-axis represents the number of patients. Insets are regression plots demonstrating the correlation between beta power and integrated EMG in exemplary cases. **P<0.01. *p<0.05. ns, non-significant. Test against zero using Wilcoxon signed-rank test. (D) A case demonstration (from subject *PD-GPi #5*) showing 30 s of simultaneously recorded chin EMG activities and basal ganglia beta power in Parkinson’s disease (PD). The first row shows the average power envelope of EMG activities filtered between 80 and 160 Hz. The second row shows the corresponding time–frequency representations of EMG activities. The third row depicts the average basal ganglia beta power filtered between 13 and 30 Hz. The fourth row shows the time–frequency representations of basal ganglia beta activities. (E) A case demonstration (from subject *DYS-GPi #2*) showing 30 s of simultaneously recorded chin EMG activities and basal ganglia beta power in dystonia. The layout of the panel is the same as in (D). (F) Beta-EMG connectivity as assessed using envelope correlation is tested against time-block shuffled surrogates (n=200 for each epoch) in three groups of patients. **P<0.01. ns, non-significant, permutation test. (G) Beta-EMG envelope correlation is compared between patients with PD and dystonia. For box plots, the lower and upper borders of the box represent the 25th and 75th percentiles, respectively. The centerline represents the median. The whiskers extend to the smallest and largest data points that are not outliers (1.5 times the IQR). **P<0.01. Mann-Whitney U test. (H) Time-lagged cross correlation between beta and EMG activities in two groups of patients with PD. The magnitude of correlation peaks at −200 ms favouring beta leading in both the PD-globus pallidus internus (GPi) and PD-subthalamic nucleus (STN) groups. The transparent grey area represents the 97.5th percentile of correlation coefficients in time-block shuffled surrogates at each temporal lag. Cross-correlation values below the grey area are considered non-significant under no correction.

We next investigated whether there existed a direct correlation relationship between beta power and EMG activities during REM sleep. On a cross subject level, we identified a significant positive correlation between average basal ganglia beta power and integrated EMG for both the GPi (p=0.003, figure 3A) and STN (p=0.010, Wilcoxon signed-rank test, figure 3B) in subjects with PD, but not in subjects with dystonia (figure 3C). This result was robust to changing a different approach (EMG variance) in quantifying EMG activities (online supplemental figure 2). On a single subject level, time-frequency analysis further indicated a potential accompanying relationship between time-resolved basal ganglia beta and EMG activities during REM sleep in PD, but not dystonia (figure 3D,E). We quantified this accompanying relationship using the approach of envelope correlation. A significant Spearman’s envelope correlation was detected between basal ganglia beta and EMG activities in subjects with PD, for both the STN (p=0.012) and GPi (p=0.013, permutation test), but not in subjects with dystonia (figure 3F,G). Notably, the magnitude of basal ganglia beta-EMG envelope correlation per se was not correlated with either the beta power (*ρ*=0.235, p=0.144) or the integrated EMG (*ρ*=0.173, p=0.286) in all subjects.

We further investigate the temporal–spatial characteristic of beta-EMG connectivity. Time-lagged cross correlation demonstrated that the average time-lagged connectivity peaked at around −200 ms favouring basal ganglia beta leading EMGs in both the PD-GPi and PD-STN groups (figure 3H). This was further corroborated by time-domain Granger causality analysis, which showed that at 200 ms lag, the probability of beta leading was significantly higher than that of EMG leading in both the GPi (G-causality for beta vs EMG, 0.12±0.07 vs 0.05±0.05, p=9.76×10^−4^) and STN (G-causality for beta vs EMG, 0.09±0.05 vs 0.04±0.03, p=2.13×10^−4^, Wilcoxon signed-rank test). Regarding the spatial distribution, results showed that basal ganglia beta-EMG envelope correlation was distributed independently of beta power in both the GPi (*ρ=−*0.02) and STN (*ρ=−*0.08) (online supplemental figure 3).

### Basal ganglia beta-EMG connectivity is associated with the clinical severity of RBD in PD

**Figure 4:**
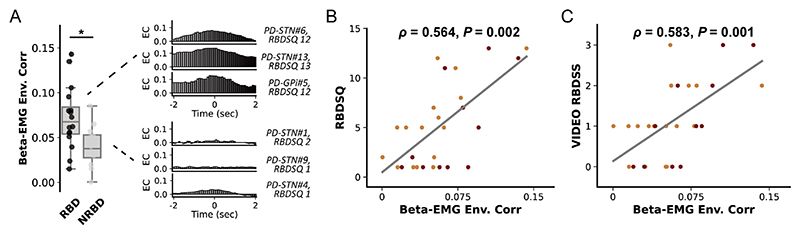
Basal ganglia beta-electromyogram (EMG) connectivity and the clinical severity of rapid eye movement (REM) sleep behaviour disorder (RBD). (A) The left plot compares the beta-EMG connectivity between patients with Parkinson’s disease (PD) with and without REM RBD. We use the 5-point threshold in RBD-Screening Questionnaire (RBDSQ) for a clinical diagnosis of RBD (scored ≥5 points). For box plots, the lower and upper borders of the box represent the 25th and 75th percentiles, respectively. The centerline represents the median. The whiskers extend to the smallest and largest data points that are not outliers (1.5 times the IQR). *P<0.05. Mann-Whitney U test. The right column shows the beta-EMG cross-correlation results and the RBDSQ score of exemplary cases with and without RBD (non-RBD, NRBD). (B) The regression plot shows the Spearman correlation between the magnitude of envelope correlation and the clinical severity of RBD as assessed by RBDSQ. The red dots represent data from the PD-globus pallidus internus (GPi) group. The orange dots represent data from the PD-subthalamic nucleus (STN) group. (C) The regression plot shows the Spearman correlation between the magnitude of envelope correlation and the clinical severity of RBD as assessed by the video-RBD severity scale. Video-RBD severity scale evaluates the severity of RBD during the recorded night by scoring both the movements and vocalisations captured during REM sleep. The red dots represent data from the PD-GPi group. The orange dots represent data from the PD-STN group.

Finally, we investigated whether basal ganglia beta activities can be associated with the severity of RBD in PD. However, we found no correlation between REM sleep beta power and the RBDSQ score for both the GPi (*ρ*=0.200, p=0.554) and STN (*ρ*=−0.026, p=0.921). In addition, integrated EMG in REM sleep was also not correlated with RBDSQ (*ρ*=0.232, p=0.235). But we observed that in PD subjects with clinically diagnosed RBD (RBDSQ ≥5), the magnitude of basal ganglia beta-EMG connectivity as assessed using envelope correlation was significantly higher than in subjects without RBD (p=0.017, Mann-Whitney U test, figure 4A). Spearman correlation revealed that beta-EMG connectivity was significantly correlated with RBDSQ score in GPi (*ρ*=0.629, p=0.038), STN (*ρ*=0.718, p=0.001) and together (*ρ*=0.564, p=0.002, figure 4B). Notably, since RBDSQ as a screening scale could have limited sensitivity in tracking RBD severity, we repeated the correlation analysis using a video-based RBD severity scale.^21^ Results revealed that beta-EMG connectivity was still significantly correlated with the video-RBD score in GPi (*ρ*=0.793, p=0.004), STN (*ρ*=0.564, p=0.018) and together (*ρ*=0.583, p=0.001, figure 4C). Importantly, this correlation was not observed in subjects with dystonia (*ρ*=0.084, p=0.796).

## Discussion

In this study, we have three main conclusions. First, basal ganglia beta power is specifically elevated during loss of atonia in REM sleep in patients with PD. Second, basal ganglia beta power increases linearly with the increase of chin EMG activities during REM sleep, with the elevation of beta power preceding the activation of muscular activities by ~200 ms. Third, the magnitude of basal ganglia-muscular connectivity correlated with the clinical severity of RBD. These findings support a prominent role of PD-related basal ganglia circuit changes in the pathophysiology of RBD.

Previous literature suggests that RBD is associated with abnormalities in brain regions including but not limited to the brainstem and motor cortex.^25^ Under normal conditions, the chin EMG amplitude progressively declines from wakefulness to NREM, and REM sleep, with the REM sleep atonia being mediated by the sublaterodorsal nucleus that is located within the dorsal pons.^12 26^ Lesions in the sublaterodorsal nucleus could result in reduced inhibition of spinal motor neurons, therefore leading to RSWA.^27^ In addition to the brainstem, abnormal activation of the motor cortex during REM sleep also contributes to RBD.^28^

Given the restored motor flexibility in patients with PD during RBD, it is proposed that the basal ganglia loop could be bypassed during RBD movement.^6 7^ The theory was further supported by several subsequent studies. Mayer *et al*^29^ using ictal single photon emission CT (SPECT) found that no metabolic change was observed within the basal ganglia region in three out of four patients during RBD episodes. Hackius’s analysis^8^ of STN electrophysiology in four patients with PD during RBD revealed a decrease in cortical-basal ganglia beta coherence, leading them to conclude that RBD-related movements bypass the cortical-basal ganglia loop. However, it is worth noting that, for the SPECT study, the limited spatial resolution of the SPECT technique may result in activations in small basal ganglia structures being undetected. And for the electrophysiological study, the decrease in cortical-basal ganglia beta coherence during movement is also expected during wakefulness,^30^ and may not be sufficient to support that the basal ganglia circuit is bypassed. On the other hand, a recent study revealed significantly enhanced basal ganglia high-frequency oscillations during RBD movements,^9^ which could be an electrophysiological marker of basal ganglia activations. Other studies linking RBD with basal ganglia showed that patients with RBD are associated with a disruption of functional connectivity^31^ and more severe dopaminergic denervation^32^ within the basal ganglia network. Our recent findings that the efficacy of DBS on RBD could be affected by the localisation of electrodes within the basal ganglia structure^33^ also support the role of basal ganglia in the pathophysiology of RBD.

Our study shows that compared with normal REM sleep, RSWA is associated with a significant increase in beta activity in the basal ganglia in PD, and beta activity can precede chin EMG activity in both the STN and GPi, which is consistent with previous observations.^8^ We did not observe such EMG-related beta elevation in NREM sleep and dystonia, suggesting that the increase of EMG activity in NREM sleep and dystonia may not have a pathophysiological basis related to RBD. In PD, the excessive beta activity in wakefulness is typically related to impaired movement, while in RBD this oscillatory pattern appears together with an ‘increased’ movement. Regarding this ‘paradox’, we have several hypotheses. (1) The increase in EMG in RSWA may be a manifestation of Parkinsonian rigidity. It has been shown that the increase of EMG in RSWA is a joint result of abnormal REM sleep and Parkinsonian rigidity in PD.^34^ This may corroborate our findings that it is mainly the low beta power (a band more related to Parkinsonian rigidity, figure 2C) that is related to loss of atonia in REM sleep episodes. However, this factor alone may not be sufficient to explain the beta elevation in RSWA. If EMG-related beta enhancement is solely due to PD rigidity, we should be able to see a similar enhancement in NREM sleep. Alternatively, or additionally, (2) beta increase during RSWA may represent microarousals, which could shift beta power towards the level of wakefulness. However, based on the literature, microarousal events in REM sleep typically occur only in severe sleep apnoea syndrome.^35^ And if the EMG-related beta elevation is due to microarousals, it should also be observed in patients with NREM sleep and dystonia, which however is not the case. (3) Previous studies have also observed that beta activity changes precede abnormal limb movements in REM sleep, and they proposed that this may reflect activation of the hyperdirect pathway, which serves to inhibit subsequent movements.^8^ We were cautious about this hypothesis. On one hand, the proposed activation of the hyperdirect pathway does not actually inhibit movement (movement gets even more flexible),^6 7^ and on the other, the beta changes we observe are mainly in the low beta band, while it is the high beta band that is generally considered to function in the hyperdirect pathway.^36^ (4) Beta activity during REM sleep may function in controlling muscle activities in REM sleep in a way that is different from wakefulness and is not yet understood.

The connectivity between basal ganglia LFP and EMG has been extensively studied in the context of tremor and gait,^22 37^ but scarcely in RBD. We found that the basal ganglia-muscular connectivity was significantly higher in PD than in dystonia, and higher in patients with PD-RBD than in patients without RBD. This connectivity was finally directly correlated with the severity of RBD. It is worth noting that this increased connectivity is not simply due to an increase in beta or EMG activity per se, as the strength of connectivity is not correlated with either beta or EMG amplitude. In addition, we did not find a direct correlation between beta power or EMG activities and the severity of RBD. Therefore, our results suggest that it is the magnitude of the connectivity between basal ganglia LFP and EMG that reflects the severity of RBD. We hypothesise that the basal ganglia beta-EMG connectivity indexes the communication between basal ganglia and limb muscles. This communication may physiologically exist during wakefulness, as evidenced by studies conducted in the context of tremor,^37^ gait^22^ and grasping.^38^ In normal REM sleep, basal ganglia-muscular communication is interrupted by the motor inhibitory effects of the brainstem nucleus, while in patients with RBD, the communication is re-established due to the loss of brainstem inhibitory functions. Since it remains unclear whether this communication is the cause or the consequence of RBD movement, it requires further investigation whether a disruption of basal ganglia-muscular communication during REM sleep through DBS or other neuromodulation techniques^39^ could lead to improved RBD symptoms.

Several limitations of our study should be highlighted. First, because the determination of the DBS target was based solely on clinical considerations, the two PD populations in the GPi and STN cohorts could have different disease profiles, such as motor severity in our study. This difference may impair comparability between the groups. Future studies should consider implementing a stricter matching procedure between groups to ensure optimal comparability. Second, to fully use recorded data, we did not exclude patients with PD based on their age at onset. Four patients with young-onset PD (age of onset<50) were included in the analysis, who could have different clinical features and basal ganglia electrophysiological profiles from late-onset PD.^40^ While since most comparisons were made using a within-subject design, its impact on the validity of conclusions should be minor. Third, event-related analysis on the dream-enactment event was not conducted due to the limited number of events captured. Data for RBD-related vocalisation were particularly scarce, as clear vocalisations were observed in only three patients with PD. This could be attributed to the low duration of RBD behaviour during total REM sleep time,^41^ coupled with the fact that patients with PD exhibit even less REM sleep. Additionally, recordings for most patients were available for only one night. Future recordings across multiple nights using sensing-enabled devices^42^ may potentially help address this issue. Fourth, we used only the chin EMG to quantify the loss of REM atonia. Although this is the standard way of diagnosing RSWA,^43^ EMG attaching also to the most affected limb may give a more comprehensive depiction of the severity of RBD.

This study finds that the basal ganglia exhibit elevations in beta power during RSWA, and the connectivity between basal ganglia beta oscillation and chin EMG activity during REM sleep is related to the clinical severity of RBD in PD. Our results support that basal ganglia activities are associated with if not directly contribute to the occurrence of RBD. This finding contributes to the understanding of the mechanism underlying RBD in PD and may aid in the development of improved DBS therapies that function by interrupting this connectivity.

## Supplementary Material

Supplementary Material

## Figures and Tables

**Table 1:** Demographics of the included patients and REM sleep descriptions of the recorded night

Patient	Dx (y)	Motor score*	PSQI	RBDSQ	RBDSS†	RSWA‡	REM sleep description
PD-GPi (n=11)							
PD-GPi 1	20	46/28	16	11	2	14.1	Proximal movements; no vocalisation
PD-GPi 2	6	53/23	9	2	0	4.8	No visible motor activity; no vocalisation
PD-GPi 3	6	33/21	13	5	2	56.2	Proximal movements; no vocalisation
PD-GPi 4	5	29/15	6	1	1	14.5	Small limb movements; no vocalisation
PD-GPi 5	9	24/4	15	12	3	39.4	Proximal and facial movements; mumbling
PD-GPi 6	8	45/31	9	1	0	13.0	No visible motor activity; no vocalisation
PD-GPi 7	10	69/31	13	7	1	34.3	Facial movements; no vocalisation
PD-GPi 8	6	71/32	6	1	0	21.6	No visible motor activity; no vocalisation
PD-GPi 9	7	45/23	5	1	1	25.0	Small limb movements; no vocalisation
PD-GPi 10	8	66/37	17	13	3	40.8	Proximal and facial movements; laughing, mumbling
PD-GPi 11	5	38/23	5	1	0	65.5	No visible motor activity; no vocalisation
Median (IQR)	7.0 (3.0)	45.0 (33.0)/23.0 (10.0)	9.0 (9.0)	2.0 (10.0)	1.0 (2.0)	25.0 (26.7)	
PD-STN (n=17)							
PD-STN 1	23	22/15	6	2	1	0.8	Small limb movements; no vocalisation
PD-STN 2	8	37/30	15	1	1	10.8	Facial movements; no vocalisation
PD-STN 3	8	34/16	9	2	0	10.9	No visible motor activity; no vocalisation
PD-STN 4	15	55/8	13	1	0	30.4	No visible motor activity; no vocalisation
PD-STN 5	7	37/18	10	11	2	8.9	Proximal and facial movements; no vocalisation
PD-STN 6	16	28/6	9	12	3	51.3	Proximal movements including violent behaviour; mumbling
PD-STN 7	8	27/5	6	5	1	26.3	Small limb movements; no vocalisation
PD-STN 8	7	24/4	8	8	1	30.0	Facial movements; no vocalisation
PD-STN 9	4	51/25	12	1	0	5.5	No visible motor activity; no vocalisation
PD-STN 10	8	25/12	10	7	1	13.3	Small limb movements; no vocalisation
PD-STN 11	8	46/17	15	5	1	43.8	Small limb movements; no vocalisation
PD-STN 12	10	28/20	10	5	1	35.5	Small limb movements; no vocalisation
PD-STN 13	8	19/4	6	13	2	75.8	Proximal movements; no vocalisation
PD-STN 14	15	42/28	4	5	1	24.8	Small limb movements; no vocalisation
PD-STN 15	11	30/18	3	1	0	16.6	No visible motor activity; no vocalisation
PD-STN 16	13	24/13	5	7	2	31.7	Proximal and facial movements; no vocalisation
PD-STN 17	5	23/17	7	6	2	9.1	Proximal movements; no vocalisation
Median (IQR)	8.0 (6.5)	28.0 (15.5)/16.0 (12.0)	9.0 (5.0)	5.0 (6.0)	1.0 (1.5)	24.8 (23.6)	
DYS-GPi (n=12)							
Dyst-1	3	M/8	10	–	1	23.0	Small limb movements; no vocalisation
Dyst-2	5	M/7	9	–	0	14.9	No visible motor activity; no vocalisation
Dyst-3	5	M/23	9	–	0	8.0	No visible motor activity; no vocalisation
Dyst-4	4	M/6	6	–	0	8.3	No visible motor activity; no vocalisation
Dyst-5	3	M/12	19	–	0	5.4	No visible motor activity; no vocalisation
Dyst-6	5	M/13	14	–	1	3.8	Small limb movements; no vocalisation
Dyst-7	7	M/16	5	–	0	1.1	No visible motor activity; no vocalisation
Dyst-8	15	M/18	11	–	0	8.9	No visible motor activity; no vocalisation
Dyst-9	3	M/9	3	–	0	4.3	Occasional loss of atonia; no vocalisation
Dyst-10	6	C/38	7	–	0	5.1	No visible motor activity; no vocalisation
Dyst-11	7	M/13	6	–	1	6.3	Small facial movements; no vocalisation
Dyst-12	3	M/16	6	–	0	5.7	No visible motor activity; no vocalisation
Median (IQR)	5.0 (3.8)	–	8.0 (4.8)	–	0.0 (0.75)	6.0 (4.3)	

* Motor score was the MDS-Unified Parkinson’s Disease Rating Scale-III OFF/ON medication for Parkinson's disease, Toronto Western Spasmodic Torticollis Rating for cervical dystonia, and Burke-Fahn-Marsden Dystonia Rating Scale (movement) for oromandibular dystonia.

† RBDSS score was obtained by visual inspection of movement and vocalisation behaviours during REM sleep (see the Materials and methods section).

‡ RSWA was the percentage of time of REM sleep without atonia among all REM sleep time. C, cervical dystonia; Dx, disease duration; Dyst, dystonia; M, Meige syndrome (oromandibular dystonia); NA, not applicable; PD, Parkinson’s disease; PSQI, Pittsburgh Sleep Quality Index; RBDSQ, REM Sleep Behaviour Disorder-Screening Questionnaire; RBDSS, REM Sleep Behaviour Disorder-Severity Scale; RSWA, REM sleep without atonia.

## Data Availability

Data are available upon reasonable request. The original data are not yet openly available, as it is being used in ongoing projects. We welcome enquires for sharing this as part of a collaboration, please contact the corresponding authors.

## What is Already Known on This Topic

⟹ Rapid eye movement (REM) sleep behaviour disorder (RBD) is an important prodromal marker and one of the most common sleep problems in Parkinson’s disease (PD). Synchronised basal ganglia beta oscillations are a hallmark of PD while its relationship with RBD remains unclear.

## What This Study Adds

⟹ This study shows that the connectivity between basal ganglia beta oscillations and chin muscular activities during REM sleep is robustly related to the clinical severity of RBD in patients with PD.

## How This Study Might Affect Research, Practice or Policy

⟹ The findings of the present study represent an expansion to the current understanding of RBD in PD and may aid in the development of improved therapies for RBD that function by interrupting pathological basal ganglia-muscular communications during REM sleep.
