# Supplementary material for: Contribution of basal ganglia activity to REM sleep disorder in Parkinson’s disease
Source: J Neurol Neurosurg Psychiatry. 2024 Apr 19;95(10):947–55. doi: 10.1136/jnnp-2023-332014 (PMC7616468; doi:10.1136/jnnp-2023-332014)
Supplement: Supplementary data [file jnnp-2023-332014supp001.pdf]

Supplementary Material

Supplementary Methods

Inclusion and exclusion criteria of patients. The inclusion criterion for Parkinson's disease (PD) was an unquestioned diagnosis based on the UK brain bank criteria. The inclusion criteria for dystonia were (i) predominantly cervical or oromandibular dystonia without prominent limb involvement (i.e., generalized dystonia patients was not included because they could not cooperate well with whole-night sleep recordings due to abnormal postures), and (ii) elderly patients with age of 40-70 years for a match with PD. The exclusion criteria for all patients included the incapacity to cooperate with polysomnography recordings and the presence of cerebral lesions on MRI scan

Beta burst dynamic analysis. Beta burst dynamics were analyzed using previously established approaches.[1,2] Data were first down-sampled to 200 Hz and decomposed using 10-cycle Morlet wavelets. For each 1-Hz frequency bin within the beta band range (18 bins, from 13 to 30 Hz), the z-scored wavelet amplitude was then obtained. A threshold was established for each bin as the 75th percentile of the wavelet amplitude among data from all hemispheres of all subjects. Beta burst was further determined when the instantaneous power exceeded the threshold for over 100 ms. For each frequency bin we extracted burst duration, amplitude, and density. The final burst dynamic descriptions were the average of all 18 iterations.

Temporal order of LFP-EMG connectivity. We used cross-correlation to investigate the temporal order between local field potential (LFP) beta and electromyogram (EMG) activities.[3] Cross correlation could be privileged over algorithms relying on Granger causality as it required less mathematical premises and can be readily implemented following envelope correlation analysis.[4] Spearman cross-correlation was estimated between whitened beta and EMG power envelops limited by 3,000 ms around each component in a 100 ms step. The correlation coefficient at each lag was tested similarly against the time-block shuffled surrogates. As a complementary, conventional time-domain Granger causality analysis was also performed to validate the directionality between brain activities. We used 500 Hz data here as a higher sampling rate helped with the Granger causality estimation. The instantaneous wavelet envelopes of beta and EMG activities were input to the algorithm *grangercausalitytests*[5] after being stationarized through first differencing. The G-causality value was estimated as the logarithm of variance difference between including and not including lagged observations in predicting time-series.[6] The G-causality values were compared between the hypotheses of beta leading and EMG leading.

Electrode reconstruction and spatial analysis. We used the advanced electrode localization pipeline with default settings in Lead-DBS version 2.5 for deep brain stimulation electrode reconstruction.[7] The Euclidean midpoint of two contacts was employed to represent the coordinate of a bipolar recording site. We projected z-scored beta power and beta-EMG envelope correlation

Yin Z, *et al. J Neurol Neurosurg Psychiatry* 2024; 95:947–955. doi: 10.1136/jnnp-2023-332014

coefficient to the surface of the GPi and STN (projection radius = 3 vertices) to investigate their respective spatial distributions. Only vertices that were touched by at least 10 data points were eligible for analysis.

Yin Z, *et al. J Neurol Neurosurg Psychiatry* 2024; 95:947–955. doi: 10.1136/jnnp-2023-332014

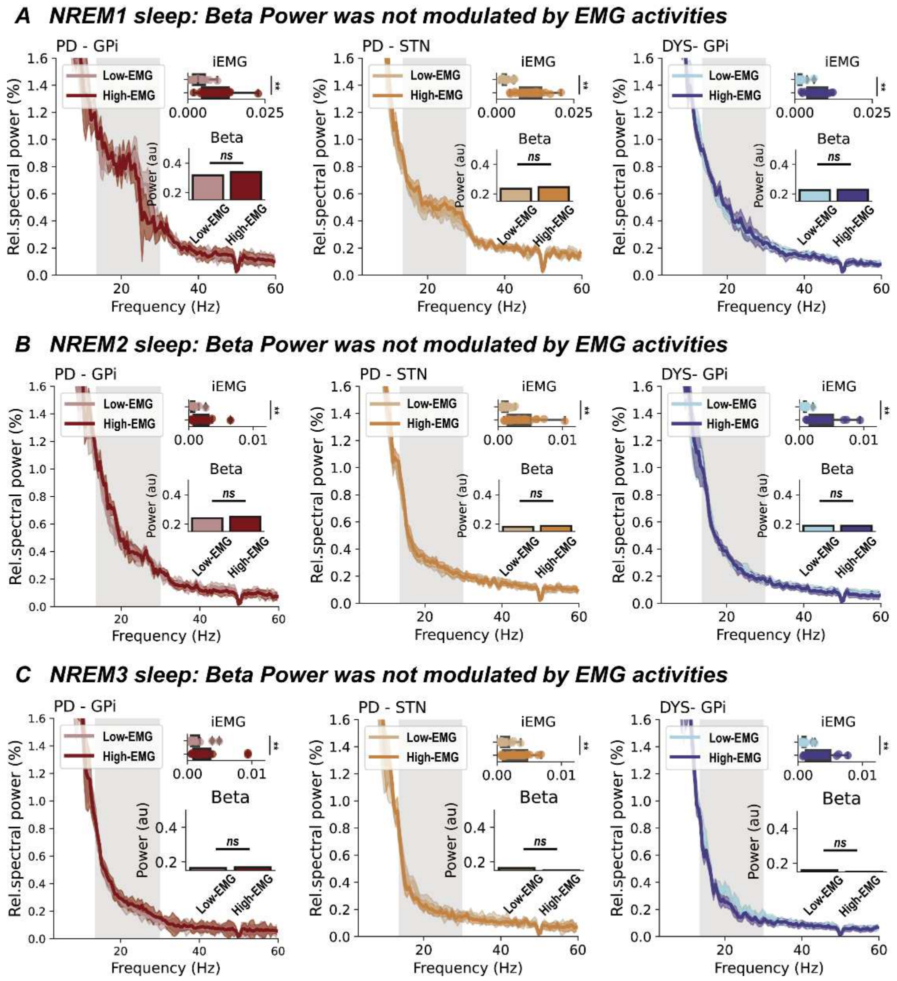

Supplementary Figure 1. Relationship between basal ganglia beta power and chin EMG activities in NREM sleep substages. (*A*) The integrated EMG, average power spectra, and beta power in non-REM stage 1 (NREM1) sleep episodes with below (low-EMG) and above median (high-EMG) EMG activities in three groups of patients. For the comparison of integrated EMG, P=9.77×10^{-4} for PD-GPi; P=1.53×10^{-5} for PD-STN; and P=4.88×10^{-4} for DYS-GPi. For the comparison of beta power, P=0.096 for PD-GPi; P=0.134 for PD-STN; and P=0.850 for DYS-GPi. (*B*) The integrated EMG, average power spectra, and beta power in NREM2 sleep episodes with below (low-EMG) and above median (high-EMG) EMG activities in three groups of patients. For the comparison of integrated EMG, P=9.76×10^{-4} for PD-GPi; P=1.53×10^{-5} for PD-STN; and P=4.88×10^{-4} for DYS-GPi. For the comparison of beta power, P=0.126 for PD-GPi; P=0.306 for PD-STN; and P=0.910 for DYS-GPi. (*C*) The integrated EMG, average power spectra, and beta power in NREM3 sleep episodes with below (low-EMG) and above median (high-EMG) EMG

Yin Z, *et al. J Neurol Neurosurg Psychiatry* 2024; 95:947–955. doi: 10.1136/jnnp-2023-332014

activities in three groups of patients. For the comparison of integrated EMG, P=1.95×10^{-3} for PD-GPi; P=1.22×10^{-4} for PD-STN; and P=1.95×10^{-3} for DYS-GPi. For the comparison of beta power, P=0.432 for PD-GPi; P=0.234 for PD-STN; and P=0.375 for DYS-GPi. Wilcoxon signed-rank test. **P<0.01. ns, non-significant.

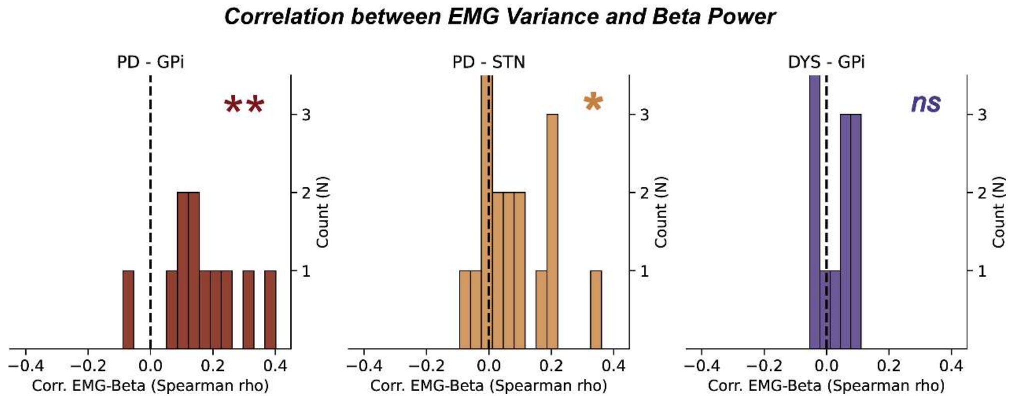

Supplementary Figure 2. Correlations between EMG variance and beta power. The distributions of episode-wise correlation coefficients between basal ganglia beta power and chin electromyogram (EMG) variance in three groups of patients. *N* in y-axis represents the number of patients. For PD-GPi (left), *P*=0.003; for PD-STN (middle), *P*=0.014; for DYS-GPi (right), *P*=0.092. Test against zero using Wilcoxon signed-rank test. ***P*<0.01, **P*<0.05, ns, non-significant.

Yin Z, *et al. J Neurol Neurosurg Psychiatry* 2024; 95:947–955. doi: 10.1136/jnnp-2023-332014

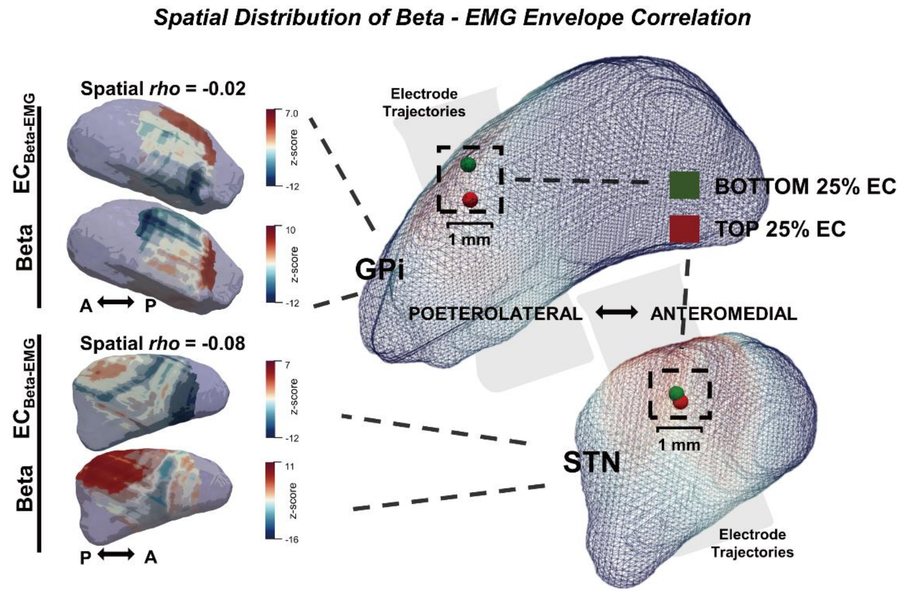

Supplementary Figure 3. Spatial distribution of the basal ganglia beta-EMG envelope correlation. The left column shows the spatial distribution of z-scored beta-electromyogram (EMG) envelope correlation (EC) and beta power on the surface of the globus pallidus (GPi) and subthalamic nucleus (STN). Warmer and cooler color indicates higher and lower z-scored values, respectively. Note that only vertices that are touched by at least 10 data points are analyzed. Areas in purple (touched by less than 10 data points) are not analyzed. The right column shows the location of the centroid of channels demonstrating bottom 25% (green) and top 25% (red) beta-EMG envelope correlation within the GPi and STN regions. The centroid of channels demonstrating top 25% connectivity was located more lateral (ΔX=0.02 mm), posterior (ΔY=0.13 mm), and superior (ΔZ=0.99 mm) in the GPi and more lateral (ΔX=0.14 mm), posterior (ΔY=0.19 mm), inferior (ΔZ=0.22 mm) in the STN than that demonstrating bottom 25% connectivity, although none of the differences reached statistical significance. Color in the nucleus' surface represents the density of passed electrodes with warmer color indicating higher implantation density.

Yin Z, *et al. J Neurol Neurosurg Psychiatry* 2024; 95:947–955. doi: 10.1136/jnnp-2023-332014

Supplementary Table 1. Medication information for the included PD and dystonia patients

Patient	Medication
PD-GPi 1	Madopar, Amantadine, Piribedil
PD-GPi 2	Madopar, Sinemet, Pramipexole
PD-GPi 3	Madopar, Piribedil, Entacapone, Pramipexole
PD-GPi 4	Madopar
PD-GPi 5	Madopar, Pramipexole, Amantadine, Sinemet
PD-GPi 6	Madopar, Piribedil
PD-GPi 7	Madopar, Sinemet
PD-GPi 8	Sinemet, Piribedil, Entacapone
PD-GPi 9	Madopar
PD-GPi 10	Madopar, Entacapone, Pramipexole
PD-STN 1	Madopar, Amantadine, Selegiline
PD-STN 2	Madopar, Pramipexole, Amantadine
PD-STN 3	Madopar
PD-STN 4	Madopar, Sinemet
PD-STN 5	Madopar, Pramipexole, Amantadine
PD-STN 6	Madopar, Pramipexole, Amantadine, Sinemet
PD-STN 7	Madopar, Pramipexole, Entacapone
PD-STN 8	Madopar, Pramipexole, Amantadine, Selegiline
PD-STN 9	Madopar
PD-STN 10	Madopar
PD-STN 11	Madopar, Pramipexole
PD-STN 12	Madopar, Pramipexole
PD-STN 13	Madopar, Pramipexole, Rasagiline
PD-STN 14	Madopar, Pramipexole, Amantadine
PD-STN 15	Madopar, Pramipexole, Entacapone
PD-STN 16	Madopar, Pramipexole, Entacapone
PD-STN 17	Madopar
Dyst-1	Clonazepam, Tiapride hydrochloride, Mecobalamin
Dyst-2	Botulin, Carbamazepine
Dyst-3	Clonazepam
Dyst-4	Baclofen
Dyst-5	Tiapride hydrochloride
Dyst-6	Tiapride hydrochloride
Dyst-7	Botulin
Dyst-8	Botulin
Dyst-9	Clonazepam

Yin Z, *et al. J Neurol Neurosurg Psychiatry* 2024; 95:947–955. doi: 10.1136/jnnp-2023-332014

Dyst-10	NA
Dyst-11	Botulin, Tiapride hydrochloride
Dyst-12	Botulin

Yin Z*, et al. J Neurol Neurosurg Psychiatry* 2024; 95:947–955. doi: 10.1136/jnnp-2023-332014

Supplementary Table 2. Comparisons of basal ganglia oscillatory features between atonia and loss of atonia REM sleep episodes

	Atonia REM sleep	Loss of atonia REM sleep	*P* value*
**PD-GPi (*n* = 11)**			
Power-Theta	0.121 (0.015)	0.107 (0.031)	0.160
Power-Beta	0.381 (0.111)	0.437 (0.160)	**0.004**
Power-Gamma	0.096 (0.066)	0.095 (0.062)	0.275
Coh-Theta	1.062 (0.050)	1.074 (0.078)	0.625
Coh-Beta	4.555 (0.081)	4.566 (0.133)	0.492
Coh-Gamma	5.473 (0.133)	5.446 (0.118)	0.695
**PD-STN (*n* = 17)**			
Power-Theta	0.152 (0.042)	0.144 (0.025)	0.074
Power-Beta	0.250 (0.080)	0.291 (0.070)	**< 0.001**
Power-Gamma	0.130 (0.092)	0.132 (0.054)	0.393
Coh-Theta	1.138 (0.107)	1.119 (0.060)	0.932
Coh-Beta	4.592 (0.207)	4.588 (0.188)	0.640
Coh-Gamma	5.473 (0.115)	5.463 (0.108)	0.580
**DYS-GPi (*n* = 12)**			
Power-Theta	0.159 (0.047)	0.159 (0.058)	0.301
Power-Beta	0.250 (0.069)	0.250 (0.041)	0.110
Power-Gamma	0.097 (0.055)	0.088 (0.043)	0.733
Coh-Theta	1.095 (0.077)	1.097 (0.184)	0.380
Coh-Beta	4.489 (0.090)	4.506 (0.127)	0.519
Coh-Gamma	5.446 (0.046)	5.524 (0.232)	0.077

Data are presented as medians (IQR).

*Statistics were obtained using Wilcoxon test. Significant comparisons were highlighted in bold.

Coh, coherence.

Yin Z, *et al. J Neurol Neurosurg Psychiatry* 2024; 95:947–955. doi: 10.1136/jnnp-2023-332014

Supplementary References

1. Tinkhauser G, Pogosyan A, Tan H, Herz DM, Kuhn AA, Brown P. Beta burst dynamics in Parkinson's disease OFF and ON dopaminergic medication. *Brain*. 2017;140(11):2968-2981. doi:10.1093/brain/awx252

2. Lofredi R, Neumann WJ, Brücke C, et al. Pallidal beta bursts in Parkinson's disease and dystonia. *Mov Disord*. 2019;34(3):420-424. doi:10.1002/mds.27524

3. Cohen MX. *Analyzing Neural Time Series Data: Theory and Practice*. The MIT Press; 2014.

4. Meyer GM, Spay C, Beliakova A, et al. Inhibitory control dysfunction in parkinsonian impulse control disorders. *Brain*. 2020;143(12):3734-3747. doi:10.1093/brain/awaa318

5. Seabold S, Perktold J. Statsmodels: Econometric and statistical modeling with python. In: *9th Python in Science Conference*. ; 2010.

6. Roebroeck A. Granger Causality. In: *Brain Mapping*. Elsevier; 2015:593-597. doi:10.1016/B978-0-12-397025-1.00337-7

7. Horn A, Li N, Dembek TA, et al. Lead-DBS v2: Towards a comprehensive pipeline for deep brain stimulation imaging. *Neuroimage*. 2019;184:293-316. doi:10.1016/j.neuroimage.2018.08.068

Yin Z, *et al. J Neurol Neurosurg Psychiatry* 2024; 95:947–955. doi: 10.1136/jnnp-2023-332014
